# Salivary Calprotectin as a Biomarker in Early Onset Inflammatory Bowel Disease: A Pilot Study

**DOI:** 10.3390/jcm14124232

**Published:** 2025-06-14

**Authors:** Simone Liguori, Gennaro Musella, Daniela Adamo, Erasmo Miele, Noemi Coppola, Federica Canfora, Carmela Del Giudice, Gianrico Spagnuolo, Sandro Rengo, Michele Davide Mignogna, Stefania Leuci

**Affiliations:** 1 Department of Neuroscience, Reproductive and Odontostomatological Sciences, University of Naples Federico II, 80138 Naples, Italy; simone.liguori@unina.it (S.L.); d.adamo@unilink.it (D.A.); noemi.coppola@unina.it (N.C.); federica.canfora@unina.it (F.C.); carmela.delgiudice@unina.it (C.D.G.); gspagnuo@unina.it (G.S.); sandro.rengo@unina.it (S.R.); mignogna@unina.it (M.D.M.); stefania.leuci@unina.it (S.L.); 2 Department of Clinical and Experimental Medicine, University of Foggia, 71122 Foggia, Italy; 3 Department of Life Science, Health, and Health Professions, Link Campus University, 00165 Rome, Italy; 4 Department of Translational Medical Science, Section of Pediatrics, University of Naples Federico II, 80138 Naples, Italy; erasmo.miele@unina.it; 5 Therapeutic Dentistry Department, Institute of Dentistry, Sechenov University, 119991 Moscow, Russia

**Keywords:** salivary calprotectin, early onset inflammatory bowel disease, prognosis, biomarkers, saliva, oral

## Abstract

**Objectives:** This study aimed to evaluate the potential of salivary calprotectin (SCP) as a novel biomarker in the management of Early Onset Inflammatory Bowel Disease (EOIBD), comparing EOIBD and healthy controls and differentiating patients based on their history of oral manifestations (OM). We correlated SCP and fecal calprotectin (FCP) in EOIBD and assessed the prognostic accuracy of SCP in predicting disease relapses. **Methods:** A sample of stimulated saliva was collected at baseline by 27 EOIBD and 9 healthy controls and then processed by ELISA for SCP determination. At sampling, a stool specimen was also provided by each patient for routine FCP assessment. Clinical disease activity was measured through Pediatric Ulcerative Colitis Activity Index (PUCAI) or Pediatric Crohn’s Disease Activity Index (PCDAI) at baseline and during follow-up at 4, 8 and 12 weeks. **Results:** A history of OM was described by 13 EOIBD. EOIBD with OM reported significantly higher SCP than EOIBD without OM (*p* < 0.01**) and controls (*p* < 0.05*). When evaluating the correlation between SCP and FCP in EOIBD with OM, positive FCP values (>120 mg/kg) were found to be associated with higher SCP concentrations (*p* < 0.05*), while in EOIBD without OM, a negative correlation was described (*p* < 0.05*). Lastly, EOIBD with OM who reported higher SCP were found to have significantly increased risk of relapse (*p* < 0.05*). **Conclusions:** In EOIBD with OM SCP was significantly more elevated and was correlated to intestinal inflammation and flare-up risk. Our results suggest the potential use of SCP as prognostic biomarker in children with intestinal and oral disease.

## 1. Introduction

Inflammatory Bowel Disease (IBD), including ulcerative colitis (UC) and Crohn’s disease (CD), is a chronic, immune-mediated condition that affects 1.03 million people in Western Europe [1].

Although IBD most frequently strikes individuals in the 2nd and 4th decade of life, children are affected as well and the incidence of Early Onset-IBD (age of onset < 17) is markedly increasing both in industrialized and developing countries [2,3,4].

Gut is not the only organ to be affected by IBD. Oral cavity is involved in the 20–50% of CD cases and in the 8% of UC cases [5,6]. Oral manifestations (OM) of IBD can be divided into specific and nonspecific based on the presence of non-caseous granulomas on histopathological examination [5]. Specific OM (i.e., lip swelling, cobblestoning, mucosal tags, and mucogingivitis) are pathognomonic of Crohn’s disease, while nonspecific OM (i.e., apthous-like ulcers, angular cheilitis, and pyostomatitis vegetans) can be triggered by nutritional deficiencies, polymicrobial infections, and medications inducing hyposalivation and topical immunosuppression, or may be the clinical sign of systemic conditions such as diabetes mellitus, primary or secondary immunodeficiencies, Sjogren syndrome, and autoimmune hepatitis [7,8,9].

The prevalence of OM is higher in children than in adults: up to 50% of pediatric patients with IBD, in fact, develops oral lesions as the primary presenting sign of CD and these lesions have been associated with upper GI tract and perianal involvement in 81% and 48% of cases, respectively [10,11]

Especially in pediatric patients, OM are often independent from intestinal disease and up to 30% of patients in clinical remission continue to suffer active oral lesions [12].

Non-invasive biomarkers have gained increasing interest in the management of several systemic diseases. Fecal calprotectin (FCP) plays a major role in the diagnostic work-up of IBD patients and in their prognostic evaluation. Several studies have reported higher levels of FCP in IBD patients than in controls, both in adults and in pediatric population [13,14] and, as a laboratory marker, FCP has revealed better diagnostic accuracy than ESR and CRP in differentiating IBD patients from healthy controls and in assessing the disease’s endoscopic and histologic activity [14,15,16,17]. Furthermore, FCP has reported relatively good sensitivity and specificity in predicting IBD relapses and patients’ response to pharmacological treatment [18]. Nonetheless FCP has some limitations. Firstly, a unique cutoff for IBD diagnosis and relapse prediction is missing. Furthermore, handling stools is perceived as dirty and embarrassing, and FCP, as a monitoring tool, is often poorly accepted by patients [18,19]. A novel, more comfortable, and equally reliable biomarker is desirable.

Calprotectin is also assayed in saliva. High levels of salivary calprotectin (SCP) have been described in several oral diseases such as gingivitis and periodontitis [20], exfoliatio areata linguae [21], oral candidiasis [22], recurrent aphthous stomatitis [23], oral cancer [24], and systemic conditions such as Sjogren syndrome [25], systemic lupus erythematosus [26], Behçet disease [27], cystic fibrosis [28], and type 1 diabetes mellitus [29] may determine increased levels of S100A8/A9 in saliva.

To the best of our knowledge this was the first study to evaluate SCP in Early Onset-IBD (EOIBD). Firstly, we compared SCP levels between healthy controls (HC), EOIBD patients with intestinal manifestations alone, and EOIBD patients with intestinal and oral involvement. Then, we assessed any correlation between SCP and clinical and laboratory data. Finally, we evaluated the potential of SCP as an indicator of disease flare-up.

## 2. Materials and Methods

### 2.1. Study Population

A pilot study was performed at the University of Naples Federico II. Ethical approval was obtained by the Ethics Committee Campania 3 (protocol number 00007974). The study was conducted according to the World Medical Association Declaration of Helsinki. Written informed consent was obtained from all participants and their parents or legal guardians.

Thirty-six subjects were included in the study. EOIBD (*n* = 27) were recruited at the Division of Pediatrics and were divided in two subgroups based on the history of OM. All EOIBD met the inclusion criteria (i.e., age < 18 years and a diagnosis of UC or CD according to established criteria [30]). Patients who assumed systemic drugs, other than IBD medications, in the previous four months, reported periodontal disease (defined as a Modified Gingival Index value ≥ 2 for more than 10% of the teeth) at oral examination or were affected by any oral or systemic disease that could influence SCP were excluded. HC (*n* = 9) were recruited at the Division of Pediatric Dentistry. The inclusion criterion was age <18 years; the exclusion criteria were systemic drugs assumption in the previous four months, periodontal disease (defined as a Modified Gingival Index value ≥ 2 for more than 10% of teeth) at oral examination, and any oral or systemic disease that could influence SCP.

### 2.2. Oral Examination and Sampling

To evaluate periodontal health and to assess OM, each participant underwent an oral examination through the use of a dental chair and a standard light. For gingival assessment, the Modified Gingival Index, that is based on visual examination of gingival tissue, was used [31], and periodontal evaluation was conducted by a single examiner to avoid inter-examiner variability.

A sample of 5 mL of stimulated whole saliva was collected from each child by spitting into a collection sterile tube after chewing on a 0.5 g paraffin tablet for 5 min, using a standardized sampling protocol [32,33,34]. Saliva was collected at 9.00 a.m. after at least one hour of food and drink restriction, as described in previous studies [21,34]. After collection samples were put on ice and stored at −80 °C until analysis. Additionally, each participant provided a stool specimen for routine FCP determination.

### 2.3. Salivary Calprotectin Determination

SCP was obtained by Enzyme-Linked Immunosorbent Assay (CALPRO SEK504Hu, Cloud-Clone Corp., Katy, TX USA). Samples were thawed at room temperature, mixed using a vortex mixer and processed on a microplate according to the manufacturer’s guidelines. Optical densities (O.D.) were then measured by reading the absorbance values at 450 nm with a microplate absorbance reader (iMARK^TM^, Bio-Rad Laboratories S.r.l., Segrate, MI, Italy). A standard reference curve was made using the standard range provided by the kit (31.2–2000 pg/mL). The concentration of calprotectin in the samples was calculated, comparing the samples’ O.D. to the standard curve to obtain SCP values as pg/mL.

### 2.4. Clinical Assessment

For each patient data about the onset, the progression and the treatment of the intestinal and oral manifestations were collected. Endoscopic disease activity was graded using the Ulcerative Colitis Endoscopic Index of Severity (UCEIS) and the Simple Endoscopic Score for Crohn’s disease (SES-CD) [35,36]. Clinical disease activity was measured, at baseline and during follow-up visits (4–8–12 weeks), through Pediatric Ulcerative Colitis Activity Index (PUCAI) and Pediatric Crohn’s Disease Activity Index (PCDAI) [37,38].

### 2.5. Statistical Analysis

Mean ± SD were used for descriptive statistics. The results of ELISA assay were presented as mean ± SEM of three independent replicates. Comparisons of categorical and quantitative non-normal demographic and clinical data were evaluated by Fisher’s Exact test and the Mann–Whitney U test, respectively. The Independent-samples *t*-test was used to assess SCP distribution among the different groups and any correlation between SCP and clinical and endoscopic activity was evaluated through Spearman’s coefficient. To investigate SCP accuracy in predicting disease flare-up, the receiver–operating characteristic curve (ROC) was made and the area under the curve (AUC) was calculated. Normality was assessed using the Shapiro–Wilk test, while variance homogeneity between data was evaluated with Levene’s test. All conducted tests were two-sided and *p*-value ≤ 0.05 (*), ≤0.01 (**), or ≤0.001 (***) were considered statistically significant. SPSS 29.0 (IBM Corporation, Armonk, NY, USA) and GraphPad Prism 10.4.0 (GraphPad Software, Boston, MA, USA) were the software used for statistical analysis and graphing.

## 3. Results

### 3.1. Demographic and Clinical Characteristics

HC were younger than EOIBD patients (HC vs. EOIBD: *p*-value < 0.01**; HC vs. EOIBD + OM: *p*-value < 0.01**). Gender and BMI were homogenously distributed among the different groups.

A total of 9 children received the diagnosis of CD, while 18 children suffered from UC. OM were more prevalent in CD than in UC although no statistically significant association was found between groups and disease type (*p*-value = 0.17).

At baseline all the patients were in treatment with immunosuppressive drugs, 19 were in clinical remission (PUCAI/PCDAI <10) and 8 had mild disease (PUCAI/PCDAI < 35).

SESCD was higher in CD with oral involvement than in CD without, but this difference was not statistically significant (*p*-value = 0.17). UCEIS, instead, was comparable between IBD subgroups.

Almost all UC patients had an extensive intestinal involvement (Montreal classification E3), only 1 patient had a localized inflammatory process (Montreal classification E1).

A total of 5 patients with CD had an ileocolonic disease (Montreal classification L2), while 4 children had an ileal involvement (Montreal classification L1). Perianal lesions were reported in 6 patients, while upper GI lesions were described only in 3 children. Upper GI involvement was always associated with perianal, ileocolonic, and oral disease. L3 and L4 phenotypes were more prevalent in patients with CD with oral involvement than in patients without, although no statistically significant correlation between groups and CD location was reported (*p*-value = 0.51). Demographic and clinical characteristics are shown in Table 1.

### 3.2. Extraintestinal and Oral Manifestations

Extraintestinal manifestations affected 5 children, including 2 patients with oral and intestinal disease. Two had articular involvement, one had sclerosing cholangitis and the last experienced venous thromboembolic disease.

A total of 13 patients (6 CD and 7 UC) had OM, of which 10 had oral involvement preceding intestinal signs: 5 children experienced the onset of oral lesions years before IBD diagnosis, 5 children few months before, and 1 child in concomitance with the onset of abdominal pain and weight loss. Only two patients experienced the onset of oral lesions a few years after IBD occurrence.

Twelve children suffered from aphthous-like manifestations characterized by generalized ulcerative lesions recurring several times a month. Three patients with CD experienced specific oral lesions. All three had lower lip swelling with deep vertical fissures associated with mucosal cobblestoning and mucogingivitis in the first patient, mucosal cobblestoning alone in the second, and palatal tag like lesions in the third (Table 1).

Patients with oral involvement experienced remission of oral lesions in about 50% of cases: in 7 out of 13 patients OM persisted. Oral disease remission was independent from the type of systemic therapy administered.

In EOIBD with OM group a higher prevalence of CD was reported compared to EOIBD without OM, although no correlation was found between OM and disease type.

### 3.3. Salivary Calprotectin Comparison Between Groups

**Figure 1:**
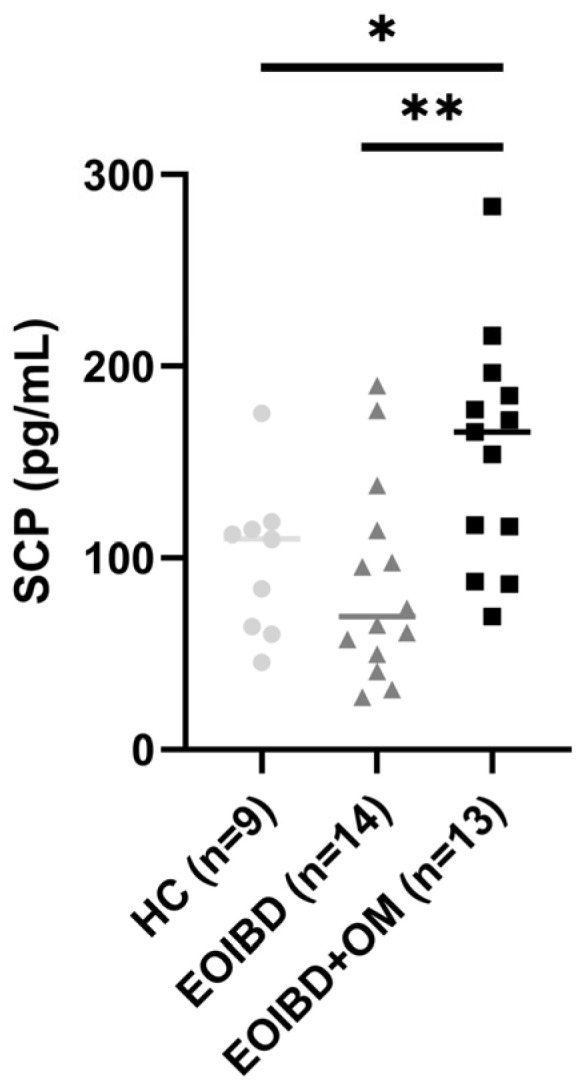
Salivary calprotectin among HC, EOIBD without OM, and EOIBD with OM. SCP, salivary calprotectin; HC, healthy controls; EOIBD, Early Onset Inflammatory Bowel Disease; OM, oral manifestations. * *p*-value < 0.05. ** *p*-value < 0.01.

When comparing SCP values between groups, no statistically significant differences were reported between EOIBD without OM and HC. EOIBD with OM reported significantly higher calprotectin concentrations than EOIBD without OM (*p*-value < 0.01**) and HC (*p*-value < 0.005*). SCP distribution among the different groups is shown in Figure 1.

### 3.4. Salivary Calprotectin and Clinical Characteristics

No statistically significant correlation was found between SCP values and disease activity, both clinical and endoscopic, in EOIBD with OM, or in EOIBD without OM groups. Furthermore, SCP levels were not associated with disease location or with the type of OM, and when stratifying patients into CD and UC, no differences in SCP values were reported based on disease type.

### 3.5. Salivary and Fecal Calprotectin Correlation

**Figure 2:**
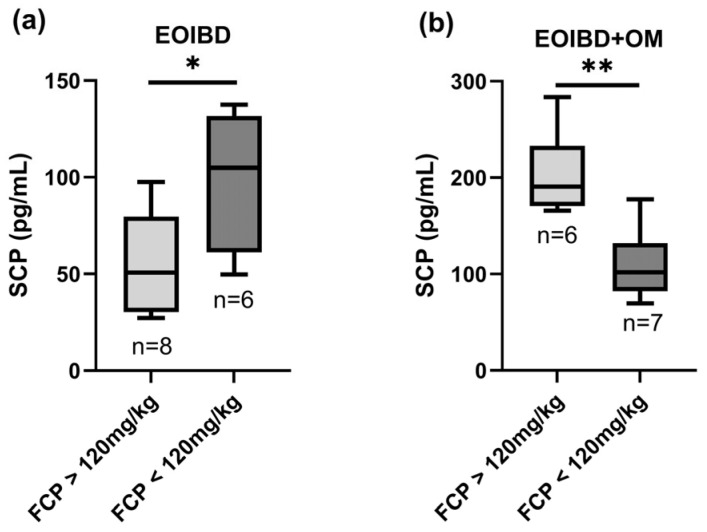
Correlation between salivary and fecal calprotectin. SCP concentration in (**a**) EOIBD without OM and (**b**) EOIBD with OM according to FCP value. SCP, salivary calprotectin; FCP, fecal calprotectin; EOIBD, Early Onset Inflammatory Bowel Disease; OM, oral manifestations. * *p*-value < 0.05. ** *p*-value < 0.01.

A statistically significant positive correlation was found when comparing SCP and FCP in EOIBD with OM (*p*-value < 0.01**); in this group, patients with FCP values > 120 mg/kg reported higher SCP, while patients with negative FCP had lower SCP. Curiously, in EOIBD without OM, a negative correlation was reported between SCP and FCP (*p*-value < 0.05*). SCP distribution according to FCP value is shown in Figure 2.

### 3.6. Salivary Calprotectin, Oral Remission, and Disease Relapse

**Figure 3:**
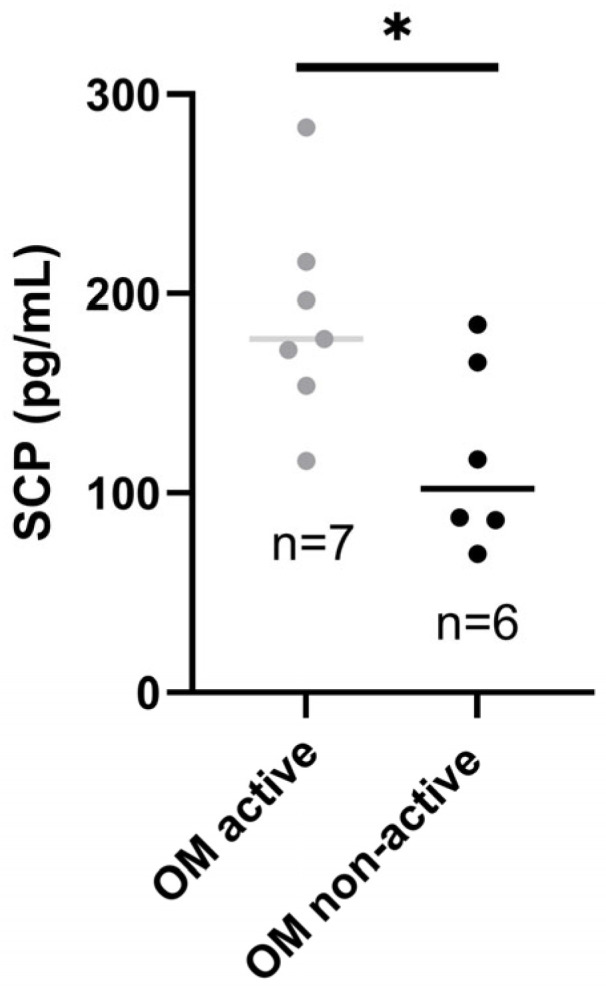
Salivary calprotectin in patients with active and inactive OM. SCP, salivary calprotectin; OM, oral manifestations. * *p*-value < 0.05.

Patients with active OM had higher SCP than those with inactive oral disease (*p*-value < 0.05*), as presented in Figure 3.

**Figure 4:**
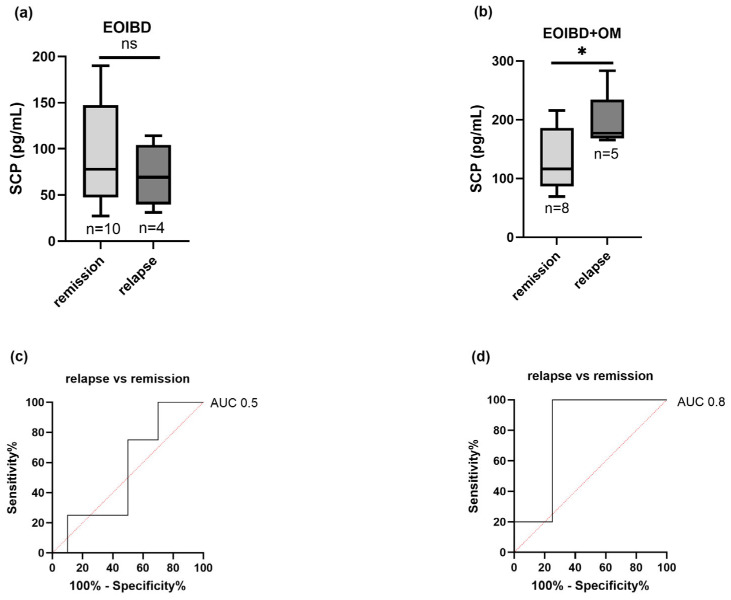
Correlation between salivary calprotectin and relapse risk. SCP concentration comparison between relapsing patients and patients experiencing remission in (**a**) EOIBD without OM and (**b**) EOIBD with OM. AU-ROC (area under receiver-operating curve) of SCP in (**c**) EOIBD without OM and (**d**) EOIBD with OM for predicting disease relapse. SCP, salivary calprotectin; EOIBD, Early Onset Inflammatory Bowel Disease; OM, oral manifestations. * *p*-value < 0.05. ns non-significant.

Nine children (four EOIBD without OM and five EOIBD with OM) experienced a relapse in the months following saliva sampling. In the EOIBD with OM group, a higher concentration of SCP was reported in relapsing patients (*p*-value < 0.05*), and when assessing SCP accuracy in predicting disease flare-up, the AU-ROC was 0.8. Contrarily, in EOIBD without OM, no correlation was reported between SCP values and relapse occurrence. Figure 4 shows the association between relapse risk and SCP in both groups.

## 4. Discussion

Calprotectin is a heterodimer formed by S100A8 and S100A9, two Ca^2+^ binding proteins, belonging to S100 family, also known as myeloid-related protein 8 (MRP8) and myeloid-related protein 14 (MRP14) or calgranulin A and calgranulin B, respectively. S100 A8/A9 is widely expressed by neutrophils, monocytes, and keratinocytes [39]. Released by immunocytes, following inflammatory stimuli, such as trauma and infection, it exerts cytokine-like functions binding to cell receptors and triggering the inflammatory pathways that modulate cell proliferation, differentiation, and migration. It also plays a direct antimicrobial action chelating zinc and manganese and thus inhibiting microbial growth and activity [15].

As other inflammatory markers, calprotectin can be assayed in multiple biologic specimens including fecal, serum, and salivary. S100 A8/A9 have been widely studied as potential biomarkers in several systemic diseases. IBD are the field of major application for calprotectin as FCP plays a pivotal role in IBD patients’ management.

Oral cavity is frequently involved in IBD as the consequence of pathobionts and inflammatory mediators’ translocation along the mouth–gut axis [40]; therefore, salivary determination of calprotectin could be particularly suitable in IBD diagnosis and prognostic monitoring.

Majster et al. assessed for the first time the concentration of SCP in patients with IBD. They reported 4.0-fold higher levels of S100A8/A9 in patients’ stimulated saliva compared to healthy controls, and SCP was found to be even more elevated in treatment naïve patients (8.2-fold higher than controls) [32]. On the contrary, the study by Bos et al. did not report any statistically significant difference between SCP in IBD and in controls, and concluded that S100A8/A9 in saliva is not a reliable biomarker to use in IBD management [41]. Additionally, both studies demonstrated no significant correlation between SCP and clinical disease activity or between salivary, serum, and fecal determination of S100A8/A9 levels.

We investigated the concentration of SCP in children with EOIBD, comparing patients based on the presence of OM. Furthermore, we evaluated SCP potential in reflecting intestinal inflammation and in predicting disease relapses.

Our results reported higher levels of SCP in EOIBD patients with oral involvement compared to controls and patients without oral lesions. We found that in children with oral disease, SCP values correlate with FCP concentrations and predict relapses occurrence, suggesting SCP potential usefulness as a prognostic non-invasive biomarker.

OM of IBD are common in pediatric patients. OM precede abdominal pain and other intestinal signs and symptoms in the 60% of cases and are more frequently reported in CD than in UC [42]. This was in line with our results that reported the presence of oral involvement in 48% of patients and a higher frequency of OM in CD than in UC.

Specific lesions of CD are less prevalent than non-specific lesions and are frequently associated with ileocolonic, upper GI, and perianal involvement [10]. Our study confirmed this association, supporting the hypothesis of a different disease subtype.

SCP levels were significantly higher in patients with OM compared to controls and EOIBD without OM. OM are, in fact, the result of abnormal immune response to oral microbiota or to the alteration of oral microbial flora after mouth colonization by gut bacteria. Both these mechanisms may lead to an increased inflammatory response and then to higher levels of salivary inflammatory biomarkers [43]. Nonetheless, our finding was in contrast with previous studies [44,45] which reported high levels of inflammatory markers in the saliva of all patients with IBD, despite their oral involvement. This difference could be explained by the fact that the non-invasive biomarkers investigated in these papers could be less accurate than calprotectin in differentiating between IBD and healthy controls. Furthermore, our study was the first to evaluate oral inflammation differentiating patients based on their OM.

Majster et al. have described a statistically significant difference between SCP levels in newly diagnosed treatment-naïve IBD and controls [32]. In our study we recruited only patients with an established diagnosis and in treatment with different immunosuppressive drugs. Nonetheless, we reported high levels of SCP in EOIBD with OM. Our finding is not in line with the papers by Bos et al. [41] and Nijakowski et al. [46] which reported lower SCP values in treated IBD patients than in healthy individuals. These authors speculated that calprotectin’s reduction in saliva could be the result of systemic host’s defence suppression mediated by immunosuppressive drugs. In agreement with previous evidence [12], we demonstrated that oral disease remission in IBD patients is independent from intestinal relief. We can assume therefore that, despite the systemic drug-mediated immunosuppression, oral host’s defence mechanisms could remain active, determining higher concentration of SCP. Furthermore, we were the first to evaluate SCP in pediatric IBD patients and this could explain the uniqueness of our findings. A different clinical phenotype has been, in fact, identified in patients with EOIBD compared to patients with late-onset disease with a more extensive location and a more rapid worsening of disease severity. Furthermore, children have been found to be less responsive to immunosuppressive therapy, and multiple therapy adjustments are often necessary to achieve clinical remission [47,48].

Our results add to the evidence that OM of IBD could mirror intestinal inflammation [49]. In our study, in fact, we reported a significant association between SCP and FCP in patients with OM, although no correlation was found between SCP and clinical or endoscopic activity.

A first limitation of our study are the small sample and the short follow-up period. This could potentially restrict the generalizability of our findings as a wider population and a longer clinical evaluation could affect the statistical significance of our research. Furthermore, a wider population group could elucidate further associations between specific clinical phenotypes, both oral and intestinal, and SCP values and a more comprehensive sample of disease spectra should be considered in future research. Nonetheless, both the size of the sample and the follow-up duration are in line with the pilot methodology of our paper that is intended to guide future examinations on this topic.

A second limitation is that we did not recruit patients with newly diagnosed IBD and thus could not investigate the diagnostic accuracy of SCP. Instead, we assessed if SCP could be suitable for prognostic evaluations. Interestingly we found higher levels in EOIBD patients with OM who experienced one or more relapses compared to patients in clinical remission. This could suggest a clinical usefulness of SCP in EOIBD patients’ follow-up.

To conclude, we found, for the first time, that in EOIBD patients with OM, SCP is significantly elevated, reflects intestinal inflammation, and can predict disease relapses. Our findings potentially suggest the use of SCP as a novel prognostic biomarker in EOIBD with OM. Nevertheless, more studies with larger samples and longer follow-up are necessary to confirm SCP’s reliability in IBD management.

## Figures and Tables

**Table 1:** Demographic and clinical characteristics.

		HC *n* = 9	EOIBD *n* = 14	EOIBD + OM *n* = 13	
Age (mean ± SD) ^a^		11.22 ± 2.22	13.57 ± 2.62	14 ± 3.24	**
Gender F/M N (%) ^b^		4/5 (44/56)	9/5 (64/36)	6/7 (46/54)	ns
BMI kg/m^2^ (mean ± SD) ^a^		21.33 ± 39.18	22.26 ± 29.05	20.97 ± 3.1	ns
SCP pg/mL (mean ± SEM) ^c^		98.21 ± 13.22	86.92 ± 13.78	155.9 ± 16.66	**
FCP mg/kg (mean ± SD) ^a^		-	605.4 ± 955.9	635.4 ± 1125.7	ns
CD N (%) ^b^		-	3 (21.4)	6 (46.2)	ns
UC N (%) ^b^		-	11 (78.6)	7 (53.8)	ns
PUCAI/PCDAI N (%) ^d^		≤5 >5	10 (71.4) 4 (28.6)	9 (69.2) 4 (30.8)	ns
UCEIS (mean ± SD) ^d^		-	3.82 ± 1.94	3 ± 1.63	ns
SES-CD (means ± SD) ^d^		-	3.67 ± 6.35	11 ± 10.65	ns
CD location N (%) ^b^	Ileal (L1) Colonic (L2) Ileocolic (L3) Upper GI (L4) Perianal (L5)	- - - - -	2 (14.3) 0 (0) 1 (7.1) 0 (0) 2 (14.3)	2 (15.4) 0 (0) 4 (30.8) 2 (15.4) 3 (23.1)	ns
UC extent N (%) ^b^	Proctitis (E1) Left colitis (E2) Pancolitis (E3)	- - -	1 (7.1) 0 (0) 10 (71.4)	0 (0) 0 (0) 7 (53.8)	ns
Oral manifestations N (%)	Aphtous-like Labial swelling Buccal cobblestoning Tags-like Mucogingivitis	- - - - -	- - - - -	12 (92.3) 3 (23.1) 2 (15.4) 1 (7.7) 1 (7.7)	
Therapy N (%) ^b^	EEN Steroids Mesalazine Methotrexate Azathioprine Infliximab Vedolizumab Adalimumab	- - - - - - - -	1 (7.1) 1 (7.1) 9 (64.3) 2 (14.3) 7 (50) 6 (42.9) 0 (0) 0 (0)	4 (30.8) 0 (0) 7 (53.8) 3 (23.1) 2 (15.4) 3 (23.1) 1 (7.7) 3 (23.1)	ns

BMI: body mass index; SCP: salivary calprotectin; FCP: fecal calprotectin; CD: Chron’s disease; UC: ulcerative colitis; PUCAI: Pediatric Ulcerative Colitis Activity Index; PCDAI: Pediatric Chron’s Disease Activity Index; UCEIS: Ulcerative Colitis Endoscopic Index of Severity; SES-CD: Simple Endoscopic Score for Crohn’s disease. - not applicable. ** *p*-value < 0.01**. ns non-significant. ^a^ Fisher’s Exact test. ^b^ Mann–Whitney U test. ^c^ Independent-samples *t*-test. ^d^ Spearman’s correlation test.

## Data Availability

The original contributions presented in this study are included in the article. Further inquiries can be directed to the corresponding author.
